# Exploration of individualized neoadjuvant therapy model for operable esophageal cancer: A Surveillance, Epidemiology, and End Results database analysis

**DOI:** 10.1002/pro6.1249

**Published:** 2024-12-08

**Authors:** Xingyu Zhou, Jiao Xue, Long Chen, Songbin Qin, Qi Zhao

**Affiliations:** ^1^ Department of Radiation Oncology The First Affiliated Hospital of Soochow University Suzhou China

**Keywords:** Esophageal cancer, Individualized therapy, Neoadjuvant therapy

## Abstract

**Purpose:**

This study aimed to examine the individualized neoadjuvant therapies for operable esophageal cancer.

**Methods and Materials:**

Data of 95,444 patients diagnosed with esophageal cancer between 2010 and 2017 were collected from the Surveillance, Epidemiology, and End Results database. The effectiveness of neoadjuvant chemoradiotherapy (nCRT), neoadjuvant chemotherapy (nCT), and surgery alone was compared in patients with stage II esophageal cancer. Patients with stage III disease were divided into “local invasive type” group (type I, T3N1M0, T4N0‐1M0) and “regional metastatic type” group (type II, T1‐2N2‐3M0) according to the tumor invasion pattern. The effectiveness of nCRT and nCT in different patterns was compared.

**Results:**

In 2,706 patients with stage II disease, a statistical difference was observed in the overall survival (OS) between nCRT (85.1%), nCT (3.0%), and surgery alone (11.9%, *P<0.001*, median OS (mOS): 54 vs 41 vs 24 months). Meanwhile, 3,303 patients with stage III disease who received nCRT were included in the propensity score matching. A statistical difference was observed in the OS between “Type I” (*n* = 217) and “Type II” (*n* = 217, *P = 0.023*, mOS: 45 VS 28 months). Among 93 patients with stage III receiving nCT, those with “Type II” (23.7%) showed a greater potential benefit from nCT than those with “Type I” (76.3%, *P = 0.686*, mOS: 51 vs 40 months).

**Conclusions:**

nCRT is recommended for stage II esophageal cancer. In patients with stage III, those with “local invasive type” may greatly benefit from nCRT, while those with “regional metastatic type” may greatly benefit from nCT.

## Graphical abstract

Neoadjuvant therapy has been widely used and achieved considerable clinical efficacy in locally advanced esophageal cancer. For stage II esophageal cancer, its stage is still early, and the degree of malignancy is between stage I and stage III. This study compared the efficacy of nCRT, nCT and surgery alone, and found that **nCRT is still recommended for stage II esophageal cancer**.

In addition, this study proposed that the tumor biological characteristics may vary according to the T and N tumor burden in esophageal cancer with the same clinical stage. Therefore, stage III operable esophageal cancer was divided into two **tumor invasion patterns** based on T and N tumor burden: **“Local invasive type”** (T3N1M0, T4N0‐1M0) and **“Regional metastatic type”** (T1‐2N2‐3M0, Figure 1).

The study explored the neoadjuvant therapy model for esophageal cancer based on different tumor invasion patterns. This study found that among stage III operable esophageal cancer, **the “Local invasive type” benefited more from nCRT, while the “Regional metastatic type” tended to benefit from nCT**. With the increasingly diversified models of neoadjuvant therapy for esophageal cancer, this finding may have guiding significance for the selection of neoadjuvant therapy model for operable esophageal cancer

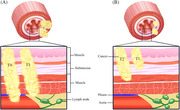

## 1 INTRODUCTION

Esophageal cancer is the sixth leading cause of cancer‐related mortality worldwide, with most patients presenting with locally advanced disease at diagnosis. Neoadjuvant therapy has emerged as a valuable approach for achieving tumor downstaging, increasing pathological complete response (pCR) rates, and improving R0 resection rates.^1^, ^2^, ^3^ These benefits collectively enhance patient survival and reduce the risk of tumor recurrence and metastasis. Accordingly, neoadjuvant chemoradiotherapy (nCRT) and neoadjuvant chemotherapy (nCT) are widely recognized as standard treatments for operable locally advanced esophageal cancer.^4^, ^5^ Additionally, neoadjuvant immunotherapy has emerged as a promising area of research, showing favorable clinical outcomes.^6^, ^7^, ^8^

The recent advancements in antitumor therapy have improved the selectivity for neoadjuvant therapies. However, individualized selection of the most effective treatment remains a challenge. A comparative evaluation of the clinical efficacy of different neoadjuvant therapies and the identification of specific patient subgroups that may most likely benefit from these treatments are crucial in making treatment decisions.

Long‐term follow‐up data from the CROSS trial demonstrated a 10‐year absolute overall survival (OS) benefit of 13% (38% versus 25%) in patients receiving nCRT.^4^ Furthermore, the 5‐year follow‐up results of the NEOCRTEC5010 trial showed an increase in the 5‐year survival rate from 49.1% to 59.9% in patients who received nCRT compared with those who underwent surgery alone.^5^ Although nCRT has become the standard treatment for locally advanced esophageal cancer, the specific subgroup that benefits most from this approach remains unclear.

However, the safety and efficacy of nCRT and nCT remain debatable. Although nCRT may improve pCR and R0 resection rates, no significant difference was found in the OS between nCRT and nCT.^9^, ^10^, ^11^, ^12^ The JCOG0909 trial suggested that nCRT may facilitate partial organ preservation and provide survival benefits, in addition to a high pCR rate.^13^ However, the CROSS trial utilized the 6th edition of the International Union Against Cancer/American Joint Committee on Cancer staging system, which only included N0/N1 staging and did not account for the number and sites of lymph node metastases. Moreover, the majority of enrolled patients had T3 and N1 disease, with only a small proportion presenting with T1–2 stage disease (17%, 63/366).^14^ However, results from the FFCD9901 trial demonstrated that patients with stage I–II esophageal cancer receiving nCRT did not experience a survival benefit and showed an increased risk of postoperative mortality.^15^

**FIGURE 1:**
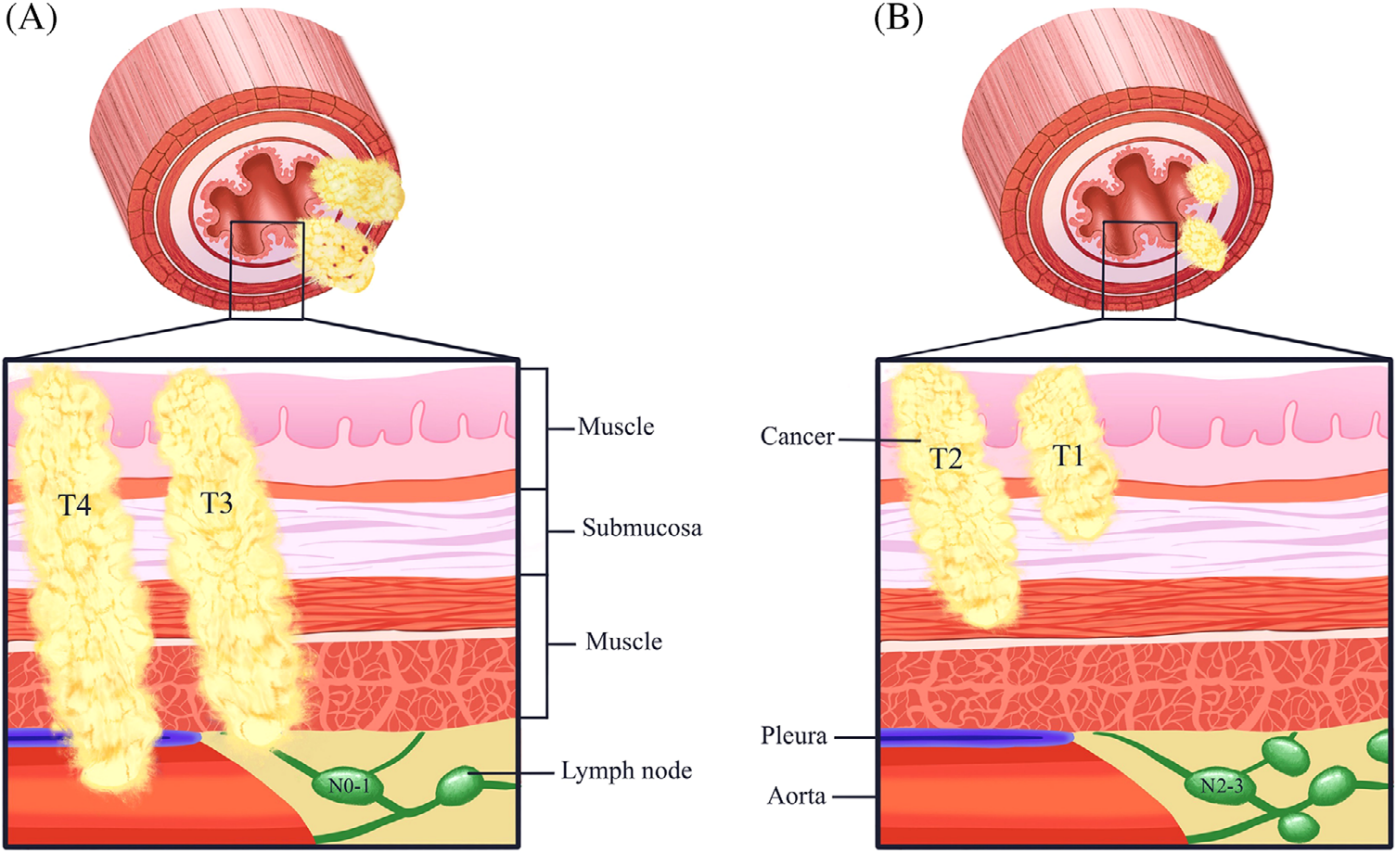
Classification of tumor invasion patterns. A. Local invasive type; B. Regional metastatic type.

Moreover, only a few studies examined the tumor invasion patterns in patients with operable esophageal cancer. In this study, we classified esophageal cancer into two distinct types based on invasion patterns: “local invasive type” (T3‐4N0‐1M0), characterized by a larger primary tumor volume and fewer regional lymph node metastases, and “regional metastatic type” (T1‐2N2‐3M0), characterized by a smaller primary tumor volume and a higher incidence of regional lymph node metastases (Figure 1).

Radiotherapy exerts a potent local effect in eradicating tumor cells, whereas chemotherapeutic drugs can target tumor cells throughout the body via the systemic circulation. Therefore, we speculate that nCRT may have a better clinical effect in patients with a “local invasive type”, while systemic treatments, such as nCT, may provide greater benefits in those with a “regional metastatic type.”

This study aimed to explore the individualized neoadjuvant therapy for stage II and III esophageal cancers. For stage III esophageal cancer, investigating the tumor invasion pattern holds great significance for the individualized selection of neoadjuvant therapies.

## 2 MATERIALS AND METHODS

### 2.1 Patients

The clinicopathological data of patients diagnosed with esophageal cancer between 2010 and 2017 were collected from the Surveillance, Epidemiology, and End Results (SEER) database (https://seer.cancer.gov/) using the SEER*Stat software (version 8.4.0.1). Patients with (1) primary tumors located in the upper, middle, or lower esophagus; (2) who underwent pathological examination to confirm the presence of clear squamous cell carcinoma or adenocarcinoma according to the International Classification of Oncology Diseases, 3rd Edition; and (3) with available data on race, age, disease stage, prognosis, and follow‐up period were included in the study. By contrast, patients (1) with TNM stage not meeting the screening criteria based on the 7th edition of the TNM staging system; (2) with stage III esophageal cancer whose staging did not meet the grouping criteria for tumor invasion patterns; and (3) whose clinical treatment did not involve surgery alone, nCRT, or nCT were excluded (Supplementary Figure 1).

### 2.2 Statistical analysis

The following data were analyzed: sex, age, race, pathological type, primary site, clinical TNM stage, survival time, survival status, chemoradiotherapy status, and surgical treatment. Survival analysis was conducted using the Kaplan–Meier method, and the log‐rank test was used to assess the impact of variables on survival outcomes. Propensity score matching (PSM) was performed to mitigate the baseline demographic differences. Univariate and multivariate Cox regression analyses were carried out to identify the potential predictors and estimate their corresponding weights. Hazard ratios (HRs) were reported as estimates along with the 95% confidence intervals (CI). All statistical analyses were performed using the IBM Statistical Package for the Social Sciences software (version 26.0).

## 3 RESULTS

### 3.1 Patients with stage II operable esophageal cancer

#### 3.1.1 Patient characteristics

Among the 2,706 patients diagnosed with stage II esophageal cancer, 2,304 (85.1%) underwent nCRT, 81 (3.0%) underwent nCT, and 321 (11.9%) underwent surgery alone. Significant differences were found in the baseline characteristics among the three treatment groups, including sex, age, race, tumor site, pathological type, and TNM stage (*P<0.001*; Table 1).

**TABLE 1:** Patient and treatment characteristics of stage II esophageal carcinoma.

Variable	nCRT+surgery (*n* = 2304)	nCT+surgery (*n* = 81)	Surgery alone (*n* = 321)	Overall (*n* = 2,706)	*p*‐value
sex, n (%)					<0.001
Male	1,839 (79.8)	64 (79.0)	204 (63.6)	2,107 (77.9)	
Female	465 (20.2)	17 (21.0)	117 (36.4)	599 (22.1)	
Age, y, n (%)					<0.001
≤60	831 (36.1)	14 (17.3)	67 (20.9)	912 (33.7)	
>60	1,473 (63.9)	67 (82.7)	254 (79.1)	1,794 (66.3)	
Race/ethnicity, n (%)					<0.001
White	2,099 (91.1)	79 (97.5)	249 (77.6)	2,427 (89.7)	
Black	85 (3.7)	1 (1.2)	12 (3.7)	98 (3.6)	
Others	120 (5.2)	1 (1.2)	60 (18.7)	181 (6.7)	
Disease site, n (%)					<0.001
Upper third	40 (1.7)	7 (8.6)	10 (3.1)	57 (2.1)	
Middle third	290 (12.6)	5 (6.2)	99 (30.8)	394 (14.6)	
Lower third	1,974 (85.7)	69 (85.2)	212 (66)	2,255 (83.3)	
Pathological type, n (%)					<0.001
Squamous	509 (22.1)	4(4.9)	179(55.8)	692 (25.6)	
Adenocarcinoma	1,795 (77.9)	77(95.1)	142(44.2)	2014 (74.4)	
Clinical TNM stage, n (%)					<0.001
T1bN1M0	103 (4.5)	1 (1.2)	67 (20.9)	171 (6.3)	
T2N1M0	642 (27.9)	26 (32.1)	59 (18.4)	727 (26.9)	
T3N0M0	1,559 (67.7)	54 (66.7)	195 (60.7)	1,808 (66.8)	

#### 3.1.2 Survival analysis

**FIGURE 2:**
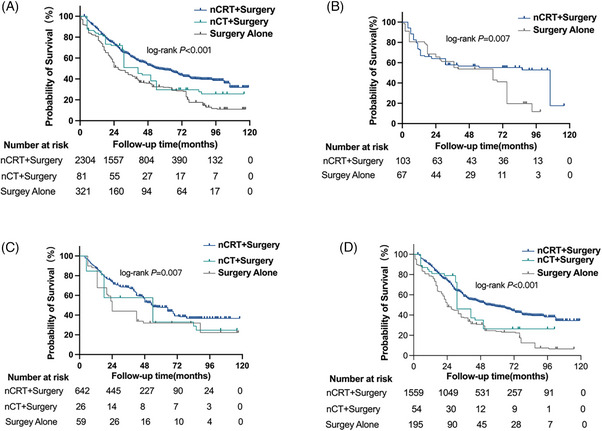
Survival curve of patients with stage II esophageal cancer. A. Stage II esophageal cancer; B. T1bN1M0; C. T2N1M0; D. T3N0M0.

Among 2,706 patients with stage II esophageal cancer, those with stage T2N0M0 were excluded from the analysis. The analysis revealed a significant difference in OS among patients treated with nCRT, nCT, or surgery alone (*P<0.001*; median OS: 54 vs. 41 months vs. 24 months). Similar conclusions were drawn when the T1bN1M0, T2N1M0, and T3N0M0 subgroups were evaluated. Notably, a small proportion of patients with T1bN1M0 tumors underwent nCT. Consequently, we compared patients who received nCRT and those who underwent surgery alone. Results showed a significant difference in OS between the two groups (*P = 0.007*, median OS: 106 months vs. 61 months). Furthermore, a significant difference was found in OS among patients with T2N1M0 disease when compared with those who received nCRT, nCT, or surgery alone (*P = 0.007*, 54 months vs. 54 months vs. 24 months). For patients with T3N0M0 disease, a significant difference was noted in OS among patients who received nCRT, nCT, or surgery alone (*P<0.001*; 53 months vs. 31 months vs. 24 months, Figure 2).

#### 3.1.3 Analysis of prognostic independent risk factors

**FIGURE 3:**
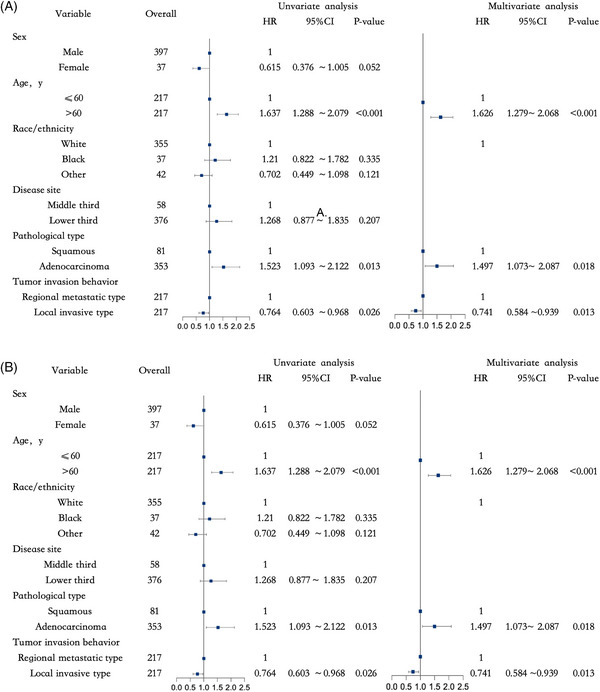
Univariate and multivariate Cox analyses. A. Stage II esophageal cancer; B. Stage III esophageal cancer treated with neoadjuvant chemoradiotherapy after propensity score matching.

Univariate Cox regression analysis identified age, sex, race, primary tumor site, pathological type, and treatment as significant factors that influenced prognosis (*P<0.05*). The subsequent multivariate Cox regression analysis identified independent prognostic factors for stage II operable esophageal cancer. The findings indicated that female patients had a better prognosis compared with male patients (*P<0.001*, HR = 0.589, 95% CI: 0.510–0.680). Patients aged >60 years demonstrated a worse prognosis compared with those aged ≤60 years (*P<0.001*, HR = 1.374, 95% CI: 1.227–1.540). The prognosis of Black patients was significantly poorer than that of White patients (*P<0.001*, HR = 1.666, 95% CI: 1.291–2.150). In terms of treatment, patients who received nCRT had a better prognosis compared with those who received nCT (*P<0.05*, HR = 0.749, 95% CI: 0.568–0.987), while patients who underwent surgery alone had a worse prognosis (*P<0.05*, HR = 1.358, 95% CI: 1.001–1.841, Figure 3A).

### 3.2 Patients with stage III operable esophageal cancer

#### 3.2.1 Patient characteristics

A total of 3,396 patients with stage III esophageal cancer and different tumor invasion patterns were enrolled. Based on the invasion patterns, the patients were classified into the “local invasive type” group (*n* = 3,157, 93.0%; T3N1M0, T4N0‐1M0) and the “regional metastasis type” group (*n* = 239, 7%; T1‐2N2‐3M0, Supplementary Table 1). The majority of esophageal cancers categorized by invasion patterns were classified as stage III. Patients with T3N0M0 were categorized as having stage II disease and excluded from the analysis. Significant differences were observed in the baseline data, including sex, age, race, tumor site, pathological type, and treatment, between the two groups (*P<0.001*). In the “local invasive type” group, 3,086 (97.8%) received nCRT, while 71 (2.2%) received nCT. In the “regional metastasis type” group, 217 patients (90.8%) received nCRT, while 22 patients (9.2%) received nCT. PSM according to tumor invasion patterns revealed 217 patients from the “local invasive type” group and 217 from the “regional metastatic type” group who had stage III operable esophageal cancer treated with nCRT. However, no significant differences were found in the baseline characteristics between the two matched groups (*P>0.05*, Table 2).

**TABLE 2:** Characteristics of patients with stage III esophageal carcinoma receiving neoadjuvant chemoradiotherapy.

	Non‐PSM			PSM		
Variable	Local invasive type (*n* = 3086)	Regional metastatic type (*n* = 217)	*p*‐value	Local invasive type (*n* = 217)	Regional metastatic type (*n* = 217)	*p*‐value
Sex, n (%)			<0.001			0.606
Male	2,559 (82.9)	200 (92.2)		197 (90.8)	200 (92.2)	
Female	527 (17.1)	17 (7.8)		20 (9.2)	17 (7.8)	
Age, y, n (%)			<0.001			0.502
≤60	2,033 (65.9)	105 (48.4)		112 (51.6)	105 (48.4)	
>60	1,053 (34.1)	112 (51.6)		105 (48.4)	112 (51.6)	
Race/ethnicity, n (%)			0.001			0.939
White	2,763 (89.5)	177 (81.6)		178 (82.0)	177 (81.6)	
Black	132 (4.3)	18 (8.3)		19 (8.8)	18 (8.3)	
Other	191 (6.2)	22 (10.1)		20 (9.2)	22 (10.1)	
Disease site, n (%)			0.061			0.397
Upper third	68 (2.2)	0 (0.0)		0 (0.0)	0 (0.0)	
Middle third	423 (13.7)	26 (12.0)		32 (14.7)	26 (12.0)	
Lower third	2,595 (84.1)	191 (88.0)		185 (85.3)	191 (88.0)	
Pathological type, n (%)			0.137			0.712
Squamous	688 (22.3)	39 (18.0)		42 (19.4)	39 (18.0)	
Adenocarcinoma	2,398 (77.7)	178 (82.0)		175 (80.6)	178 (82.0)	

#### 3.2.2 Survival analysis

**FIGURE 4:**
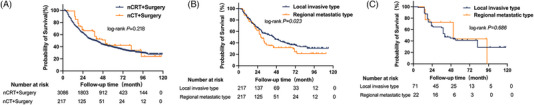
Survival curve of patients with stage III esophageal cancer. A. Patients treated with nCRT or nCT; B. Patients treated with nCRT with different invasion types after propensity score matching; C. Patients treated with nCT with different tumor invasion types.

No significant difference was observed in the OS among patients with stage III operable esophageal cancer treated with nCRT or nCT (*P = 0.218*, median OS: 39 months VS 51 months, Figure 4A). In patients treated with nCRT, a statistical difference was found in the median OS between the “local invasive type” group and “regional metastatic type” group (*P = 0.023*, median OS: 45 months vs. 28 months) after PSM. In patients receiving nCT, no statistical difference was observed in the median OS between the “local invasive type” group and “regional metastatic type” group. However, the “regional metastatic type” group exhibited a longer median survival (*P = 0.686*, median OS: 40 months vs. 51 months, Figure 4B‐C).

#### 3.2.3 Analysis of prognostic independent risk factors

After PSM of patients with stage III esophageal cancer receiving nCRT, univariate Cox regression analysis identified age, pathological type, and tumor invasion pattern as factors associated with prognosis (*P<0.05*). These factors were subsequently included in the multivariate Cox regression analysis to determine the independent prognostic factors for patients with stage III operable esophageal cancer receiving nCRT. Patients aged >60 years had a worse prognosis than those aged ≤60 years (*P<0.001*, HR = 1.626, 95% CI: 1.279–2.068). Adenocarcinoma was associated with a worse prognosis compared with squamous cell carcinoma (*P<0.001*, HR = 1.497, 95% CI: 1.073–2.087). Furthermore, the survival benefits in patients with the “local invasive type” was greater than that in those with the “regional metastatic type” (*P = 0.013*, HR = 0.741, 95% CI: 0.584–0.939, Figure 3B).

## 4 DISCUSSION

The management options for locally advanced operable esophageal cancer have significantly expanded with the development of multidisciplinary treatment approaches and novel anticancer drugs. A previous meta‐analysis demonstrated that neoadjuvant radiotherapy alone resulted in a modest improvement in the 5‐year absolute survival rate from 15% to 18%,^16^ rendering it obsolete. With regard to nCT, the safety and short‐term efficacy of the three‐drug DCF and four‐drug FOLT combination regimens have been validated alongside the traditional two‐drug CF regimen. These findings have been incorporated into the National Comprehensive Cancer Network (NCCN) guidelines.^17^, ^18^ The use of nCRT has resulted in significant improvements in patient survival compared with surgery alone, as evidenced by follow‐up data from the CROSS and NEOCRTEC5010 trials.^4^, ^5^ In the context of neoadjuvant immunotherapy, the PALACE‐1 trial demonstrated a high pCR rate of 55.6% when nCRT was combined with pembrolizumab.^7^ Similarly, the DANTE trial reported a pCR rate of 49% in patients treated with FOLT combined with atezolizumab; patients with programmed death ligand1 (PD‐1) combined positive score (CPS) >10 treated with a combination of chemotherapy and immunotherapy achieved a pCR rate of 67%.^19^

Locally advanced esophageal cancer is characterized by significant heterogeneity, encompassing a range of tumor stages from T1bN**^+^** to T4aN_any_, as defined by the Chinese Society of Clinical Oncology (CSCO) guidelines. Given the diverse permutations of available neoadjuvant therapies, the selection of an appropriate treatment for patients with locally advanced operable esophageal cancer necessitates an individualized approach. In addition to comparing the efficacy of different treatment modalities, identifying specific patient subgroups that would significantly benefit from each neoadjuvant treatment modality remains essential.

The CROSS trial established the efficacy of nCRT in the treatment of locally advanced esophageal cancer. However, the trial did not specifically identify the patient subgroup that would benefit the most from nCRT. In the CROSS trial, the majority of patients had T3 tumors (78%) and regional lymph node involvement (N1, 75%), but the number and location of lymph node metastases were not specified according to the N stage.^4^ Conversely, the FFCD9901 study demonstrated that nCRT did not improve the OS in patients with stage I or II disease.^15^ Stage I esophageal cancer has shown a better prognosis with surgery alone, whereas stage III esophageal cancer has demonstrated benefits from nCRT. The efficacy of neoadjuvant therapy for stage II esophageal cancer, which lies between stages I and III in terms of malignancy, remains controversial. This study included 2,706 patients with stage II esophageal cancer, but excluded those with a T2N0M0 stage, which is primarily indicated for surgery alone according to the CSCO and NCCN guidelines.^20^ These results suggest that nCRT is the primary recommended treatment for stage II esophageal cancer, except in patients with a T2N0M0 stage.

Only a few studies have investigated the tumor invasion patterns in esophageal cancer. Esophageal cancer is classified into two groups based on tumor invasion patterns: “ local invasive type” and “regional metastatic type.” This classification is based on various T (primary tumor) and N (metastatic lymph nodes) loads in the tumor (Figure 1). Radiotherapy is a localized treatment modality that uses radiation to eradicate tumor cells. Therefore, nCRT is thought to improve the local control rate and ultimately enhance patient survival in locally invasive cancers. On the contrary, chemotherapy is a systemic treatment approach that employs chemically synthesized drugs to kill or inhibit the growth of tumor cells throughout the body. Hence, patients with regional metastasis may benefit more from nCT.

For patients receiving nCRT or nCT, the effect of tumor invasion patterns on prognosis remains uncertain. This study included 3,396 patients with stage III esophageal cancer to evaluate the influence of tumor invasion patterns on the prognosis of patients undergoing nCRT. Significant differences were observed in OS between the “local invasive type” and “regional metastatic type” groups (*P = 0.023*, median OS: 45 months vs. 28 months, Figure 4A). Multivariable Cox regression analysis demonstrated that the tumor invasion pattern was an independent prognostic factor for stage III esophageal cancer treated with nCRT. Notably, patients with a “local invasive type” exhibited a greater survival benefit (*P = 0.013*, HR = 0.741, 95% CI: 0.584–0.939, Figure 3B).

This study also investigated the prognostic impact of tumor invasion patterns in patients undergoing nCT. No statistical difference was found in the median OS between the two tumor invasion patterns. However, patients with the “regional metastatic type” demonstrated a longer median survival (*P = 0.686*, median OS: 40 months vs. 51 months, Figure 4B). These findings support the hypothesis that tumor invasion patterns may guide neoadjuvant treatment selection in stage III esophageal cancer.

Although radiation therapy is an effective method for eradicating tumor cells, it inevitably damages the surrounding normal tissues. In esophageal cancer, radiotherapy is linked to varying degrees of radiation toxicity, affecting quality of life and prognosis in patients. The common toxicities include radiation esophagitis, and lung and myocardial injuries. Additionally, ionizing radiation can induce immunosuppression by depleting lymphocytes, potentially increasing the neutrophil‐to‐lymphocyte ratio, a marker linked to poor outcomes in multiple solid tumors.^21^ The severity of these side effects is strongly influenced by factors such as radiation dose, irradiated volume, and fractionation method. In cases of regionally metastatic esophageal cancer that require larger radiation fields, these toxic effects may partly explain the reduced benefits of nCRT.

The comparison between the efficacy and safety of nCRT and nCT has received considerable attention in esophageal cancer management. This study suggests that patients with stage II esophageal cancer who receive nCRT experience greater treatment benefits (Figure 3A). The benefits of nCRT and nCT appear to be similar (Figure 4C). Previous studies have consistently demonstrated that nCRT can further improve R0 resection and pCR rates compared with nCT.^10^, ^11^

Traditionally, surgery has been the primary treatment for esophageal cancer. However, it is associated with postoperative complications and mortality. Notably, approximately one‐third of the patients achieved pCR after receiving nCRT, suggesting the feasibility of omitting surgery through a wait‐and‐see strategy. For patients who achieve a clinical complete response (cCR) after neoadjuvant therapy, a close follow‐up regimen combined with salvage surgery is the preferred treatment strategy. This approach has been shown to enhance the quality of life without compromising OS. In the JCOG0909 trial, which included 96 patients with esophageal cancer who initially declined surgery and received concurrent chemoradiation for observation, those with a poor treatment response underwent salvage surgery. The results revealed a substantial survival benefit, with a 5‐year OS rate of 64.5% observed in two‐thirds of the patients.^22^ Given the similar survival benefits of nCT and nCRT, nCRT may offer a higher cCR rate compared with nCT, providing more patients with opportunities for organ preservation and improved quality of life.

The following conclusions were drawn from the findings of this study: 1. nCRT remains the recommended neoadjuvant therapy for stage II esophageal cancer. 2. In stage III esophageal cancer, tumor invasion patterns can guide the selection of neoadjuvant treatments. The “local invasive type” is likely to benefit more from nCRT, whereas nCT may be more beneficial for the “regional metastatic type.”

This study is among the first to consider esophageal cancer tumor invasion patterns when selecting individualized neoadjuvant therapies. Notably, the SEER database only includes data of patients from North America and lacks information on gene‐targeted therapy and immunotherapy, which may affect patient prognosis. Furthermore, the number of patients receiving nCT in the subgroup was limited, and PSM was not performed in patients with stage III esophageal cancer receiving nCT. In the era of immunotherapy, various neoadjuvant therapies, including immunotherapy combined with radiotherapy and chemotherapy, are being explored and hold promise for enhancing survival benefits in patients with esophageal cancer. However, the individualized selection of neoadjuvant therapies for esophageal cancer remains challenging and requires further investigation.

## AUTHOR CONTRIBUTIONS

**XingYu Zhou**: Software; validation; formal analysis; and writing—original draft. **Jiao Xue**: investigation; resources; and data curation. **Long Chen**: visualization; project administration; and writing—reviewing and editing. **Qi Zhao**: Conceptualization; methodology; writing—reviewing and editing; supervision; and funding acquisition.

## CONFLICT OF INTEREST STATEMENT

The authors declare that they have no competing financial interests or personal relationships that may have influenced the work reported in this study.

## ETHICAL STATEMENT

Not applicable.

## Supporting information



Supporting Information

## Data Availability

The data that support the findings of this study are available from the corresponding author upon reasonable request.
