# Supplementary material for: Exploration of individualized neoadjuvant therapy model for operable esophageal cancer: A Surveillance, Epidemiology, and End Results database analysis
Source: Precis Radiat Oncol. 2024 Dec 8;8(4):218–26. doi: 10.1002/pro6.1249 (PMC11934889; doi:10.1002/pro6.1249)
Supplement: Supplementary file 1 — Supporting Information [file PRO6-8-218-s001.docx]

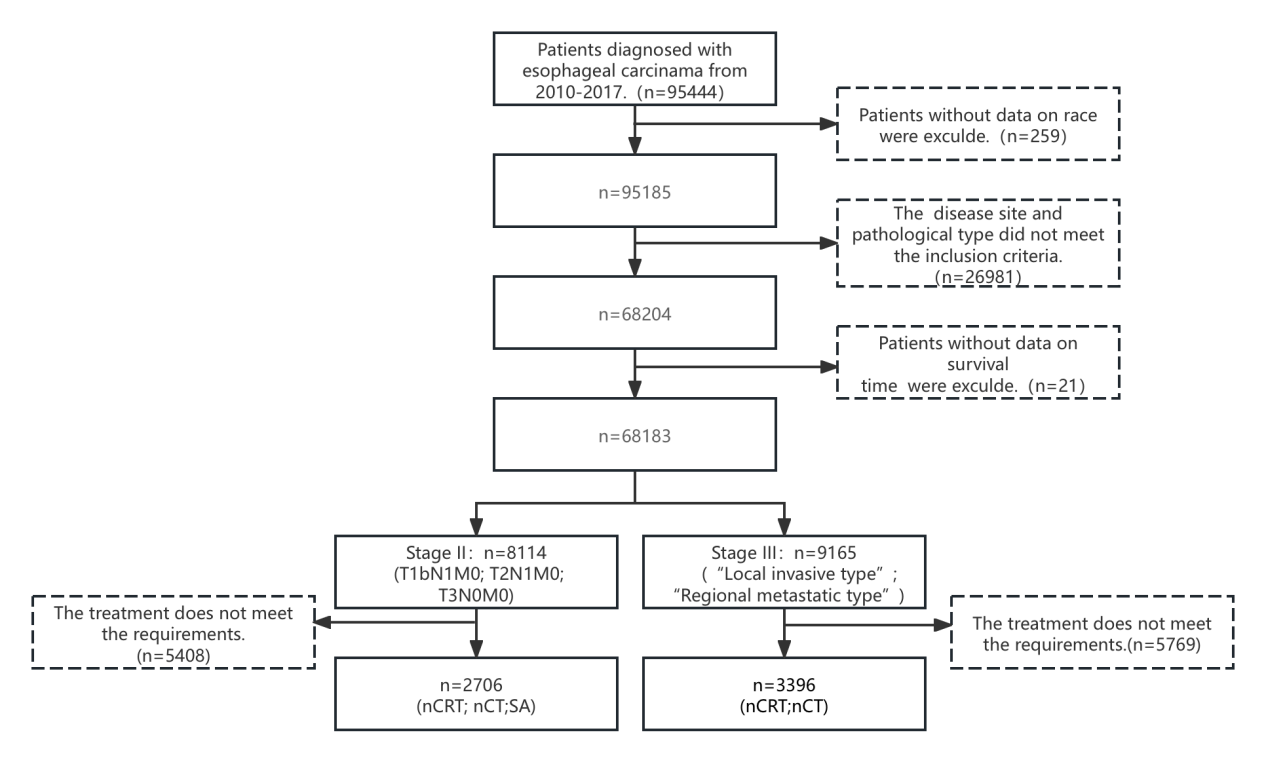


**Supplemental Fig. 1.** Data screening process.

**Supplemental Table 1.** Clinical features and treatment data of stage III esophageal cancer.

| Variable | Local invasive type（n=3157） | Regional metastatic type（n=239） | P-Value |
| --- | --- | --- | --- |
|  |  |  |  |
| Sex，n（%） |  |  | <0.001 |
| Male | 2610(82.7) | 222(92.9) |  |
| Female | 547(17.3) | 17(7.1) |  |
| Age，y，n（%） |  |  | <0.001 |
| ≤60 | 1066(33.8) | 109(45.6) |  |
| >60 | 2091(66.2) | 130(54.4) |  |
| Race/ethnicity,n(%) |  |  | <0.001 |
| White | 2832(89.7) | 189(79.1) |  |
| Black | 134(4.2) | 22(9.2) |  |
| Other | 191(6.1) | 28(11.7) |  |
| Disease site,n(%) |  |  | 0.28 |
| Upper third | 70(2.2) | 0(0) |  |
| Middle third | 429(13.6) | 26(10.9) |  |
| Lower third | 2658(84.2) | 213(89.1) |  |
| Pathological type,n(%) |  |  |  |
| Squamous | 706(22.4) | 45(18.8) | 0.204 |
| Adenocarinoma | 2451(77.6) | 194(81.2) |  |
| Treatment,n(%) |  |  | <0.001 |
| nCRT+Surgery | 3086（97.8） | 217（90.8） |  |
| nCT+Surgery | 71（2.2） | 22（9.2） |  |
